# Editorial: Advances in drug development, residues management and emerging contaminants

**DOI:** 10.3389/fvets.2026.1913978

**Published:** 2026-07-31

**Authors:** Maria Vittoria Varoni, Nicola Pugliese, Noemi Romagnoli

**Affiliations:** 1 Dipartimento di Medicina veterinaria, Università degli Studi di Sassari, Sassari, Italy; 2 Dipartimento di Medicina Veterinaria, Università degli Studi di Bari Aldo Moro, Valenzano, Bari, Italy; 3 Dipartimento di Scienze Mediche Veterinarie, Alma Mater Studiorum-Università di Bologna, Ozzano dell'Emilia, Bologna, Italy

**Keywords:** animal science, contaminants, pharmacology, residues, toxicology

In the last decades, veterinary pharmacology and toxicology have moved from the margins to the foreground of medical science.

Born as an offshoot of human pharmacology, and mostly characterized by empiricism, veterinary pharmacology and toxicology emerged as distinct and independent disciplines in the middle of 20th century, fostered by the rapid development of antimicrobials, antiparasitic, and other veterinary pharmaceutical specialties (1, 2). Their growth was driven by the need to understand species-specific differences across a diverse range of animals in terms of drug efficacy, toxicity, and pharmacokinetics, along with the species-specific response to potentially toxic substances. Nowadays, these disciplines extend beyond the treatment of individual animals to encompass food and feed safety, residues and contaminants, antimicrobial resistance, environmental risk, comparative medicine, and One Health, in order to achieve consumer safety in food-producing animals, discover new substances, new formulations, and even new approaches, tailored to the peculiarities of veterinary medicine (1, 3).

This Research Topic is heading in that direction, either presenting novel approaches to the preparation of veterinary formulations of well-known pharmaceutical compounds. It is the case of Taylor et al., who created 3D-printed gabapentin chewable tablets for small animals. Using a gelatin-based veterinary excipient (CuraVet^®^), they tested whether printing formulations could be reused after 14 days of refrigerated storage. They observed that the formulation containing the lower concentration-maintained printability and dosing precision, while that with the higher concentration developed crystals and became unstable. This study demonstrates the potential of reusable 3D-printing formulations for rapid, on-demand veterinary medicines.

Similarly, great attention has been dedicated to antitumoral chemotherapy, which has become one of the most dynamic areas of veterinary pharmacology. Recent studies aim to develop and validate specific strategies for cancer treatment in companion animals (4, 5), paving the way for innovative approaches in veterinary oncology. The toxic effects of chemotherapeutic agents are well documented (6). Although, these drugs are designed to selectively target rapidly proliferating cancer cells, they also affect healthy tissues with a high cell turnover, resulting in tissue damage and the development of toxic effects, which may be severe. On this basis, the safety of antineoplastic agents is becoming even more important. Yeh et al., reported vincristine overdose management in a Siamese cat with lymphoma that accidentally received four times the intended vincristine chemotherapy dose. Treatment included intravenous lipid emulsion, therapeutic plasma exchange, followed by attempted hemoperfusion and combined supportive care, suggesting a role of extracorporeal therapies in managing chemotherapeutic overdoses in veterinary patients.

The presence of drug residues and contaminants is a major concern in Toxicology (1). Drug residues, such as antibiotics and hormones, may persist in animal tissues if the required withdrawal periods before slaughter or milking are not respected. In addition, the inappropriate use of antimicrobials has contributed to the emergence of antimicrobial-resistant bacteria, posing a serious global public health threat. Contaminant residues, on the other hand, result from exposure to environmental and chemical substances that accumulate in soil, water, and air, with which animals may come into contact. In recent years, particular attention has been given to contaminants such as polychlorinated biphenyls (PCBs), dioxins, heavy metals, mycotoxins, and other hazardous compounds that can contaminate food. Some contaminants, such as heavy metals, can enter the food chain and eventually reach humans. Others, including mycotoxins and pesticide residues, may contaminate crops and pose toxic risks to both animals and humans. Antimicrobial resistance is responsible for thousands of deaths each year and leads to increased healthcare costs and significant economic losses due to reduced productivity. In recent years, attention has focused not only on the presence of drug and contaminant residues in foods of animal origin and their implications for consumer safety, but also on the toxicological quality of feed intended for both companion and farmed animals (7, 8). Öztürk et al. analyzed 29 chicken-based cat kibbles using different methodologies for contaminants including antibiotics, perfluoroalkyl substances (PFAS), toxic metals, bisphenol A (BPA), and microplastics. BPA and microplastics were detected in all samples, toxic metals were found in nearly all products, while antibiotic residues and PFAS were detected in some samples. Although, contaminant concentrations were generally low, chronic exposure to contaminant mixtures may pose long-term health risks to cats. Consequently, the researchers emphasize the need for stricter monitoring and comprehensive safety assessments of pet food ingredients.

The increasing reports of animal intoxications, intentional or accidental, require attention from clinical veterinarians together with the availability of new treatment strategies. Intentional poisoning involves the deliberate placement of toxic baits containing poisonous substances with the aim of causing intoxication or death in domestic and wild animals. Kim et al. reported a forensic veterinary case in South Korea involving a free-roaming cat that died from poisoning caused by ethylene glycol (EG). Necropsy and histopathological analysis revealed severe renal injury with calcium oxalate crystal deposition, which is characteristic of EG toxicity. Toxicological GC–MS analysis detected both EG and glycolic acid in gastric tissue, definitively confirming exposure. This case underscores the importance of forensic veterinary pathology and toxicology in suspected animal cruelty cases.

Accidental poisoning in animals can result from drug overdoses, the use of medications intended for other species, or the ingestion/exposure to harmful substances left unattended. This includes human medications [e.g., non-steroidal anti-inflammatory drugs (NSAIDs) and antibiotics] household and garden chemicals (e.g., herbicides, molluscicides, and antifreeze), as well as foods that are toxic to animals (e.g., chocolate, xylitol, and others). Chaganti and Brainard evaluated the adsorption capacity of three activated charcoal formulations, charcoal powder, proprietary granular or suspension formulations, and a novel resin-bound activated charcoal product against several common veterinary toxicants, including medicaments as naproxen (NSAIDs used to treat mild to moderate pain and inflammation), ivermectin (medication used to treat infection caused by parasites in human and animals), EG (a toxic substance used as antifreeze and particularly appealing to animals because of its sweet taste), baker chocolate (a food especially dangerous for pet because it contains very high concentrations of the methylxanthines theobromine and caffeine, compounds that dogs and cats metabolize much more slowly than humans) and xylitol (an artificial sweetener that is highly toxic to animals, particularly dogs because it triggers a rapid and excessive release of insulin). All charcoal formulations reduced concentrations of the assayed toxicants under both acidic and neutral conditions, although their efficacy varied depending on the compound involved. Activated charcoal was less effective against compounds such as ethylene glycol and xylitol, while granular formulations and powdered activated charcoal often acted faster than the new resin-bound product.

All of these reflect the increasing attention paid to animal welfare, which has become a central point in the everyday practice of veterinarians, and a key topic in Veterinary Medicine schools, thus inspiring the new veterinary challenge for the near future: to achieve a sustainable, One Health-oriented, and evidence-based approach capable of embracing such societal expectations and providing trustworthy options to increasingly informed and demanding stakeholders.
